# COVID-19 versus seasonal influenza: myocardial injury and prognostic importance

**DOI:** 10.1186/s12879-022-07488-y

**Published:** 2022-06-13

**Authors:** Lars Mizera, Monika Zdanyte, Johannes Gernert, Álvaro Petersen-Uribe, Karin Müller, Meinrad Paul Gawaz, Simon Greulich, Dominik Rath

**Affiliations:** Department of Cardiology and Angiology, University of Tübingen, Otfried-Müller-Str.10, 72076 Tübingen, Germany

**Keywords:** COVID-19, Prognosis, Seasonal influenza, Cardiovascular disease

## Abstract

**Background:**

Acute myocardial injury is associated with poor prognosis in respiratory tract infections. We aimed to highlight the differences in prevalence of myocardial injury and its impact on prognosis in patients with COVID-19 compared to those with seasonal influenza.

**Methods:**

This was a single-center prospective cohort study with a historical control group. 300 age-/sex-matched SARS-CoV-2 and seasonal influenza positive patients were enrolled. Myocardial injury was assessed by electrocardiogram (ECG), transthoracic echocardiography and biomarkers including high-sensitivity troponin-I. All patients were followed-up for 30 days after enrollment for all-cause mortalitiy, admission to the intensive care unit (ICU) and mechanical ventilation.

**Results:**

Right ventricular distress was more common in COVID-19 whereas pathological ECG findings and impaired left ventricular function were more prevalent among influenza patients. COVID-19 patients suffered from a higher percentage of hypertension and dyslipidaemia. Contrary to COVID-19, pericardial effusion at admission was associated with poor outcome in the influenza group. Severe course of disease and respiratory failure resulted in significantly higher rates of ICU treatment and mechanical ventilation in COVID-19 patients. Although distribution of myocardial injury was similar, significantly fewer cardiac catheterizations were performed in COVID-19 patients. However, number of cardiac catheterizations was low in both groups. Finally, 30-day mortality was significantly higher in COVID-19 compared to influenza patients.

**Conclusions:**

In adults requiring hospitalization due to COVID-19 or seasonal influenza, cardiovascular risk factors and signs of myocardial distress differ significantly. Furthermore, cardiovascular comorbidities may impair prognosis in COVID-19 patients to a higher degree than in their influenza counterparts.

## Background

Both influenza virus and severe acute respiratory syndrome coronavirus 2 (SARS-CoV-2) are RNA viruses and infect the respiratory epithelium. Aggravation of preexisting cardiovascular and respiratory comorbidities may favor a fatal outcome in influenza and SARS-CoV-2 infections [1, 2]. Extrapulmonary manifestations and cardiac involvement are common in patients hospitalized with seasonal influenza and coronavirus disease 2019 (COVID-19) [3, 4]. Furthermore, recent studies provide increasing evidence of advanced age and pre-existing cardiovascular diseases being associated with severe course of disease [2, 5]. A study by Covino et al. showed, however, that in patients aged ≥ 80 years and severe dementia were associated with poor outcome rather than increasing age itself. We and others have previously shown, that impaired myocardial function and elevated concentrations of cardiac biomarkers are associated with worse prognosis in COVID-19 [6–8]. SARS-CoV-2 promotes a pro-coagulant environment by inducing platelet activation and inhibiting fibrinolysis [9–12], leading to thromboembolic complications including deep vein thrombosis, pulmonary embolism and myocardial infarction.

In the current study, we compare the clinical course and outcomes in patients hospitalized with COVID-19 and seasonal influenza. We focus on the incidence and possible pathomechanisms of myocardial injury and respiratory failure to provide further evidence in terms of risk factors and their implications for prognosis.

## Methods

### Study design and participants

This is a prospective study with a historical control cohort. Between March 2020 and January 2021, we enrolled 441 consecutive SARS-CoV-2 positive patients, that were admitted to the University Hospital of Tübingen, Germany. The historical control cohort consisted of 285 influenza patients admitted to the University Hospital of Tübingen between December 2015 and February 2019. Influenza patients were matched according to age and sex, resulting in two patient groups consisting of 150 individuals each.

Cardiovascular risk was assessed by electrocardiogram (ECG), transthoracic echocardiography (TTE) and high-sensitivity cardiac troponin-I (hs-cTnI) levels. Myocardial injury was defined as elevated serum hs-cTnI level above 99th percentile according to the Fourth Universal Definition of Myocardial Infarction [13]. At our laboratory, the 99th percentile of hs-cTnI was 57 ng/mL for men and 37 ng/mL for women. Patients < 18 years were not enrolled in the current study.

### Diagnosis of Influenza, SARS-CoV-2 infection and ARDS

Seasonal influenza and SARS-CoV-2 infection were diagnosed from nasopharyngeal secretions using a real-time reverse transcriptase polymerase chain reaction. Severity of acute respiratory distress syndrome (ARDS) was further graduated according to the Berlin Definition of Acute Respiratory Distress Syndrome [14].

### Follow-up

All patients were followed-up for 30 days after study enrollment. The primary combined endpoint consisted of all-cause mortality and/or mechanical ventilation. All-cause mortality, mechanical ventilation or admission to intensive care unit (ICU) served as secondary endpoints. Follow-up was conducted via hospital discharge letters and telephone interviews.

### Statistical analysis

SPSS version 26.0 (SPSS Inc., Chicago IL) was applied for all statistical analyses. Normally and non-normally distributed data were compared with Student’s t-test and Mann–Whitney *U* test, respectively. Hence, values are either presented as mean ± standard deviation or median with 25th/75th percentile. Categorial variables were compared with cross tabulations and consecutive chi-squared tests. Correlations of non-normally distributed data were assessed using the Spearman’s rank correlation coefficient (rho). Kaplan–Meier curves including log rank tests were applied to compare intergroup survival. Where indicated, Cox-regression analyses were used to determine independent associations.

## Results

Baseline characteristics and incidence rates per 100 person years, both stratified according to COVID-19 and influenza, are shown in Table 1. SARS-CoV-2 infected individuals displayed increased rates of arterial hypertension and dyslipidemia whereas significantly more influenza patients actively smoked.

**Table 1:** Baseline characteristics and (IR) per 100 person years (PY) stratified according to infectious disease

	COVID-19	Influenza	
	(n = 150)	(n = 150)	*p* value
Age, years (mean ± SD)	67.8 (± 15)	67.7 (± 15)	0.962
Male, n (%)	94 (62.7)	88 (58.7)	0.478
Cardiovascular risk factors, n (%)			
Arterial hypertension	107 (71.3)	91 (60.7)	0.051
Dyslipidemia	52 (35.9)	29 (19.3)	**0.001**
Diabetes mellitus	37 (24.8)	35 (23.3)	0.591
Current smoker	7 (4.8)	29 (19.3)	** < 0.001**
Obesity	38 (26.4)	35 (23.3)	0.544
Atrial fibrillation	36 (24.2)	29 (19.3)	0.312
COPD	8 (5.3)	14 (9.3)	0.184
Immunosuppression	11 (7.4)	19 (12.7)	0.128
Coronary artery disease	35 (23.3)	47 (31.3)	0.120
Chronic kidney disease	19 (12.7)	17 (11.3)	0.880
Symptoms at admission, n (%)			
Dyspnea	81 (55.5)	57 (38.8)	**0.008**
Cough	83 (56.1)	100 (68.0)	**0.035**
Fever	92 (63.4)	72 (49.0)	**0.013**
Echocardiography			
LVEF%, mean (± SD)	57 (± 8)	54 (± 11)	0.082
Impaired LVEF, n (%)	17 (13.5)	20 (24.7)	**0.040**
Left ventricular hypertrophy, n (%)	88 (71.5)	56 (70.0)	0.813
Visually estimated impaired RV-function, n (%)	17 (13.9)	14 (17.9)	0.111
Right ventricular dilatation, n (%)	54 (45.0)	23 (29.1)	**0.024**
TAPSE, mm, mean (± SD)	22 (± 5)	21 (± 4)	0.065
PAPsys, mmHg, mean (± SD)	29 (± 11)	27 (± 12)	**0.022**
Aortic valve stenosis > 1, n (%)	3 (3.6)	6 (7.6)	0.261
Aortic valve regurgitation > 1, n (%)	7 (5.7)	2 (2.5)	0.283
Mitral valve regurgitation > 1, n (%)	22 (18.0)	17 (21.5)	0.542
Pulmonal valve regurgitation, n (%)	75 (79.2)	22 (31.9)	** < 0.001**
Tricuspid valve regurgitation > 1, n (%)	22 (18.5)	10 (12.8)	0.292
Pericardial effusion, n (%)	60 (48.4)	5 (6.2)	** < 0.001**
Electrocardiography			
Rate, bpm, mean (± SD)	84 (± 23)	87 (± 21)	0.101
Sinus rhythm, n (%)	102 (81.0)	123 (82.6)	0.779
QRS, ms, mean (± SD)	94 (± 20)	95 (± 19)	0.545
Regular R progression, n (%)	71 (58.7)	91 (61.1)	0.689
Right bundle branch block, n (%)	10 (8.2)	22 (14.9)	0.087
Left bundle branch block, n (%)	3 (2.4)	22 (14.9)	** < 0.001**
PQ segment, ms, mean (± SD)	170 (± 89)	164 (± 29)	0.218
QTc, ms, mean (± SD)	379 (± 77)	376 (± 54)	0.186
Negative T wave, n (%)	22 (18.2)	55 (37.2)	**0.002**
ST segment depression, n (%)	10 (8.2)	32 (21.6)	**0.001**
ST segment elevation, n (%)	0 (0)	2 (1.4)	0.199
Laboratory values at admission median (25th/75th percentile)			
Leucocytes, 1000/µl	6.5 (4.6/9.7)	6.7 (5.1/9.1)	0.573
Lymphocytes, 1000/µl	0.8 (0.6/ 1.1)	0.9 (0.6/ 1.4)	0.107
Haemoglobin, mg/dl	12.7 (11.1/14.1)	13.3 (12.0/14.1)	**0.044**
Platelets, 1000/µl	184 (147/244)	177 (141/220)	0.118
Creatinin, mg/dl	0.9 (0.7/1.3)	1.0 (0.8/1.3)	0.409
GFR, ml/m2	74 (50/92)	71 (49/88)	0.417
D-dimers, µg/dl	1.4 (0.7/3.0)	0.9 (0.5/1.5)	0.136
C-reactive protein, mg/dl	8.2 (2.6/16.1)	2.8 (1.5/6.6)	** < 0.001**
Procalcitonin, ng/ml	0.1 (0.1/0.9)	0.2 (0.1/1.0)	0.068
Troponin I, ng/dl	18 (6/65)	30 (30/40)	** < 0.001**
NT pro-BNP, ng/l	473 (141/3245)	1156 (160/6661)	0.421
CK, U/l	149 (74/347)	130 (72/295)	0.418
Bilirubin	0.7 (0.5/1.1)	0.5 (0.4/0.7)	** < 0.001**
AP, U/l	68 (52/88)	70 (53/92)	0.811
AST, U/l	43 (27/70)	37 (24/77)	0.529
ALT, U/l	32 (21/48)	24 (16/35)	** < 0.001**
LDH, U/l	336 (230/446)	218 (186/280)	** < 0.001**
Lactate	1.3 (1.0/1.9)	1.4 (1.0/1.9)	0.687
pH	7.43 (7.39/7.46)	7.41 (7.36/7.44)	**0.007**
Medication at admission, n (%)			
Oral anticoagulation	21 (15.7)	20 (14.3)	0.716
ACEi	32 (23.9)	49 (35.0)	**0.044**
ARB	45 (33.6)	20 (14.3)	** < 0.001**
Aldosterone inhibitors	17 (12.7)	14 (10.0)	0.483
Diuretics	51 (38.3)	53 (37.9)	0.934
Calcium channel blockers	31 (23.3)	35 (25.0)	0.744
Beta blockers	56 (41.8)	66 (47.1)	0.373
Statins	50 (37.3)	45 (32.1)	0.369
ASS	34 (25.6)	40 (28.6)	0.576
P2Y12 inhibitors	3 (2.3)	7 (5.0)	0.232
Clinical course			
Admission ICU, n (%)	77 (51.3)	15 (10.0)	** < 0.001**
First Horovitz index in mmHg, mean (± SD)	259 (± 145)	226 (± 163)	0.351
Horovitz index nadir in mmHg, mean (± SD)	190 (± 112)	119 (± 62)	**0.038**
Mechanical ventilation, n (%)	68 (45.3)	8 (5.3)	** < 0.001**
Vasopressors, n (%)	63 (56.8)	9 (60.0)	0.812
Viral coinfection, n (%)	9 (7.8)	5 (33.3)	**0.003**
Bacterial coinfection, n (%)	44 (38.3)	10 (66.7)	**0.036**
Dialysis, n (%)	21 (46.7)	4 (26.7)	0.174
ECMO, n (%)	6 (15.4)	3 (20.0)	0.684
Cardiac catheterization, n (%)	6 (4)	18 (12)	**0.011**
PCI, n (%)	4 (66.7)	9 (50.0)	0.478
Severity of ARDS, n (%)			
None	52 (34.7)	100 (66.7)	** < 0.001**
Mild	35 (23.3)	42 (28.0)	0.26
Moderate	39 (26.0)	2 (1.3)	** < 0.001**
Severe	24 (16.0)	6 (4.0)	**0.002**

Endpoints (COVID-19/Influenza)	No. of events (COVID-19/ Influenza)	IR/100 PY (COVID-19/ Influenza)	P
Combined endpoint	82 (69/13)	328 (552/104)	** < 0.001**
Mechanical ventilation	77 (69/8)	308 (552/64)	** < 0.001**
ICU admission	92 (77/15)	368 (616/120)	** < 0.001**
All-cause mortality	33 (24/9)	132 (192/72)	**0.006**

**Fig. 1:**
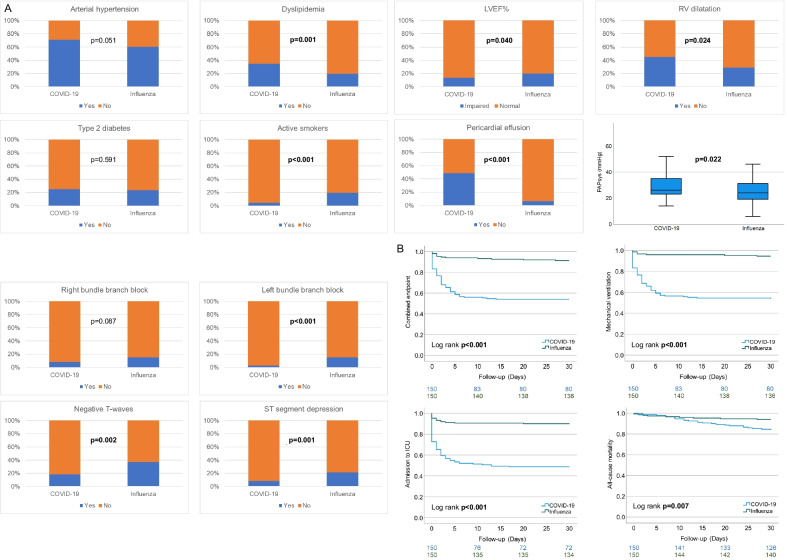
**A** Distribution of cardiovascular risk factors, echo- and electrocardiographic parameters in COVID-19 vs influenza patients. **B** Prognosis in COVID-19 vs influenza patients

Patients suffering from influenza presented with lower left ventricular function (LVEF%) at study inclusion when compared to COVID-19. On the other hand, right ventricular (RV)-function was significantly worse in SARS-CoV-2 positive patients. Fittingly, systolic pulmonary arterial pressure (PAPsys) was higher and significant pulmonary as well as tricuspid valve regurgitation were more common in these individuals. Pericardial effusion (PE) was significantly associated with occurrence of the combined endpoint in influenza patients (p < 0.001), although prevalence of PE was low compared to substantially higher rates of PE in COVID-19 patients (Fig. 1).

Pathologic ECG signs, especially left bundle branch block and ST segment depression were more prevalent in the influenza group.

Hs-cTnI was significantly elevated in influenza patients compared to SARS-CoV-2. Of note, 70 patients (37 COVID-19 vs 33 seasonal influenza, p = 0.273) in the overall cohort had an indication for cardiac catheterization based on significantly elevated hs-cTnI levels. Rate of cardiac catheterization was low in both groups, however, significantly more cardiac catheterizations were performed in influenza patients compared to COVID-19 (54.5% vs 16.2%, p = 0.011).

Rate of moderate to severe ARDS was significantly elevated in the COVID-19 group (Table 1).

Consequently, incidence rates of combined endpoint, mechanical ventilation, admission to ICU and all-cause mortality were significantly higher in COVID-19 patients compared to their influenza counterparts (Fig. 1). Of note, SARS-CoV-2 infection was independently associated with the combined endpoint, mechanical ventilation and admission to ICU, respectively. COVID-19 was the strongest independent predictor for all-cause mortality but failed to show statistical significance (Table 2).

**Table 2:** Cox Regression with epidemiological factors as independent and the combined endpoint, mechanical ventilation and all-cause mortality as dependent variables

Combined endpoint	p value	HR	95% CI
Age	0.219	0.982	(0.953−1.011)
Gender	0.566	0.795	(0.363−1.740)
Arterial hypertension	0.055	2.930	(0.975−8.803)
Dyslipidemia	0.140	0.536	(0.234−1.228)
Type 2 diabetes mellitus	0.946	1.030	(0.441−2.407)
Active smoking	0.882	0.886	(0.180−4.358)
Obesity	0.779	0.884	(0.372−2.101)
Impaired LVEF%	0.664	1.246	(0.463−3.352)
PAPsys	0.406	1.014	(0.981−1.048)
COVID-19 vs. influenza	** < 0.001**	0.139	(0.047−0.412)

Mechanical ventilation	p value	HR	95% CI
Age	0.252	0.983	(0.953−1.013)
Gender	0.344	0.678	(0.303−1.516)
Arterial hypertension	0.112	2.385	(0.815−6.974)
Dyslipidemia	0.149	0.541	(0.235−1.246)
Type 2 diabetes mellitus	0.950	1.028	(0.437−2.414)
Active smoking	0.953	1.049	(0.212−5.183)
Obesity	0.933	0.963	(0.405−2.292)
Impaired LVEF%	0.547	1.358	(0.502−3.672)
PAPsys	0.435	1.014	(0.980−1.048)
COVID-19 vs. influenza	** < 0.001**	0.100	(0.030−0.328)

ICU admission	p value	HR	95% CI
Age	0.535	0.991	(0.962−1.021)
Gender	0.148	0.560	(0.255−1.228)
Arterial hypertension	0.193	1.972	(0.709−5.485)
Dyslipidemia	0.073	0.480	(0.216−1.071)
Type 2 diabetes mellitus	0.550	1.269	(0.581−2.774)
Active smoking	0.824	0.836	(0.172−4.056)
Obesity	0.834	1.091	(0.482−2.470)
Impaired LVEF%	0.794	1.138	(0.432−2.999)
PAPsys	0.428	1.013	(0.982−1.045)
COVID-19 vs. influenza	** < 0.001**	0.126	(0.044−0.366)

All-cause mortality	p value	HR	95% CI
Age	0.967	1.001	(0.949−1.056)
Gender	0.943	1.047	(0.296−3.706)
Arterial hypertension	0.181	4.857	(0.480−49.143)
Dyslipidemia	0.364	0.554	(0.155−1.980)
Type 2 diabetes mellitus	0.127	2.594	(0.762−8.825)
Active smoking	0.981	0.000	(0.000−NA)
Obesity	0.693	0.758	(0.192−2.998)
Impaired LVEF%	0.402	1.877	(0.430−8.188)
PAPsys	0.997	1.000	(0.947−1.056)
COVID-19 vs. influenza	0.079	0.237	(0.047−1.184)

Bold indicates that *P* < 0.001 is considered independently associated to the endpoint

## Discussion

The major findings of the current study are: (1) Cardiovascular risk factors were more prevalent in hospitalized COVID-19 patients compared to influenza. (2) COVID-19 was associated with RV-distress while influenza patients presented with higher rates of impaired LV-function and ECG abnormalities. (3) A low number of patients with significantly elevated hs-cTnI levels received cardiac catherization, abandoning recommendations of the current guidelines on treatment of AMI. (4) In the current age- and sex-matched cohort, mechanical ventilation and ICU treatment were 6-times higher in the COVID-19 group and (5) SARS-CoV-2 patients had a threefold increased mortality risk when compared to individuals suffering from influenza.

Our findings confirm current evidence showing higher mortality and morbidity in SARS-CoV-2 compared to seasonal influenza [15–20]. We have previously shown that elevated PAPsys, most probably due to elevated pulmonary vascular resistance, caused by alveolar and vascular damage, leads to RV-distress in COVID-19. Consequently, elevated cardiac biomarkers are common findings in these patients [7, 8]. On the contrary, LV-dysfunction is more common in influenza, confirming the findings by *Erden et al* [21]. Although high prevalence of PE was observed in COVID-19 patients, it was only associated with adverse outcomes in influenza.

Numerous potential mechanisms leading to myocardial injury in seasonal influenza and SARS-CoV-2 infection are discussed to date. In addition to direct viral invasion, platelet hyperactivity, endothelial activation, oxygen supply and demand mismatch as well as enhanced atherosclerotic plaque vulnerability may be enhanced [22, 23]. Thus, myocardial injury due to thromboembolism may represent a cornerstone for poor prognosis in COVID-19. An increased alveolar-arterial oxygen gradient due to ventilation-perfusion mismatch or an altered alveolar diffusion barrier could be an early indicator for the necessity of oxygen supply and severe couse of disease in these patients and should be investigated in future studies, especially in contrast to influenza patients.

In the current study, cardiac catheterization was performed less frequently in COVID-19 patients compared to those suffering from influenza, which may have had an impact on increased mortality in COVID-19 patients. Thus, abandoning guideline-established treatment strategies highlights an important problem in infectious diseases.

## Limitations

Our study is limited by the single center retrospective assessment of hospitalized patients with seasonal influenza. However, a low burden of influenza infections during the COVID-19 pandemic impedes a prospective approach with large numbers of cases. One of the limitations of the study is a moderate study population size. Furthermore, validation cohorts are needed to confirm the distinct associations of COVID-19 and influenza on prognosis. Therefore, we are currently cooperating with university hospitals in Germany to exchange our data on COVID-19 and influenza. A major objective of this collaboration is to establish validation cohorts for the discovered single-centre findings. Finally, vaccination status was also not recorded in the influenza group, so a vaccination-related bias for a milder course of disease would be conceivable.

## Conclusion

In summary, clinical course, cardiac involvement and prognosis among hospitalized patients with seasonal influenza and COVID-19 differ considerably. In our opinion, acute and pre-existing cardiovascular disease affects COVID-19 patients in a far more drastic manner than their influenza counterparts, rendering stringent cardiovascular assessment and treatment by a COVID-19 heart team indispensable.

## Data Availability

The datasets analysed as part of the present study are available and can be provided by the corresponding author on reasonable request.

## Abbreviations

ARDS: Acute respiratory distress syndrome
COVID-19: Coronavirus disease 2019
ECG: Electrocardiogram
hs-cTnI: High-sensitivity cardiac troponin-I
ICU: Intensive care unit
LV: Left ventricle
RV: Right ventricle
SARS-CoV-2: Severe acute respiratory syndrome coronavirus 2
PAPsys: Systolic pulmonary arterial pressure
PE: Pericardial effusion
TTE: Transthoracic echocardiography
